# Retraction Note: Heat transfer analysis of Cu and Al_2_O_3_ dispersed in ethylene glycol as a base fluid over a stretchable permeable sheet of MHD thin-film flow

**DOI:** 10.1038/s41598-026-69210-1

**Published:** 2026-09-02

**Authors:** Zeeshan, Ilyas Khan, Wajaree Weera, Abdullah Mohamed

**Affiliations:** 1 Department of Mathematics and Statistics, Bacha Khan University Charsadda, Charsadda, KP Pakistan; 2 Department of Mathematics, College of Science Al-Zulfi, Majmaah University, 11952 Al-Majmaah, Saudi Arabia; 3 Department of Mathematics, Faculty of Science, Khon Kaen University, Khon Kaen, 40002 Thailand; 4 Research Centre, Future University in Egypt, New Cairo, 11835 Egypt

Retraction of: *Scientific Reports* https://doi.org/10.1038/s41598-022-12671-x, published online 25 May 2022

The Editors have retracted this Article.

After publication, concerns were raised regarding the authorship of this Article. The Editors requested the code, underlying raw data, and evidence for author contributions, but the Authors did not provide the data and were not able to address the concerns related to the authorship. The Editors have therefore lost confidence in the contents of this Article.

The Authors did not respond to the Editors' correspondence about this retraction.

